# Nanobodies Selectively Binding to the Idiotype of a Dengue Virus Neutralizing Antibody Do Not Necessarily Mimic the Viral Epitope

**DOI:** 10.3390/biom13030551

**Published:** 2023-03-17

**Authors:** Monica Poggianella, Robert Bernedo, Sandra Oloketuyi, Ario de Marco

**Affiliations:** 1 Molecular Immunology Laboratory, International Centre for Genetic Engineering and Biotechnolgy, Padriciano 99, 34149 Trieste, Italy; poggiane@icgeb.org; 2 Laboratory for Environmental and Life Sciences, University of Nova Gorica, 5000 Nova Gorica, Slovenia; robert.navarro@vib-ugent.be (R.B.); oloketuyisandra@gmail.com (S.O.)

**Keywords:** nanobodies, dengue, anti-idiotypic antibodies, vaccination, immune response, panning, paratope structure

## Abstract

Vaccination against dengue virus is challenged by the fact that a generic immune response can induce antibody-dependent-enhancement (ADE) in secondary infections. Only some antibodies targeting a quaternary epitope formed by the dimerization of the virus protein E possess sufficient neutralizing capacity. Therefore, the immunization with anti-idiotypic antibodies of neutralizing antibodies might represent a safe vaccination strategy. Starting from a large pre-immune library, we succeeded in isolating a wide set of anti-idiotypic nanobodies characterized by selective and strong binding to the paratope of the neutralizing antibody 1C10. However, the mice immunized with such constructs did not produce effective antibodies, despite at least some of them eliciting an immune response selective for the nanobody variable regions. The results suggest that complex conformational epitopes might be difficult to be recreated by anti-idiotypic structures. The selection process of the anti-idiotypic candidates might be optimized by applying epitope mapping and modeling approaches aimed at identifying the key residues that is necessary to bind to trigger selective immune response.

## 1. Introduction

Antibodies (Abs) recognize antigens through the combined structure formed by the heavy (H) and light (L) chains, (indicated as VH and VL, respectively), while H-chain-only Abs, such as those of camelids, do so by means of the single variable domain of the H chain (VHH). The idiotype (Id) of an Ab has been classically defined as the distinctive structure formed by the association of the two variable domains VH and VL, whereas the specific region directly interacting with the epitope on the antigen (Ag) is referred as the paratope. Usually, Abs that recognize the Id of a defined Ab (target antibody) are called anti-idiotypic Abs [1,2]. Anti-Id Abs can inhibit binding of the target antibodies to the cognate Ag with different degrees of strength, depending on the interaction modality and on the relative number of residues of the paratope belonging to the highly variable complementarity determining regions (CDRs) or to the relatively conserved framework (Fw) regions within the V domains. As a consequence, Abs directed exclusively against CDR residues of the target Ab are usually very selective because the binding involves residue combinations that are highly specific. In a limited number of cases, the interaction of anti-Id Abs with the target Ab will resemble that of the Ag with the same Ab. This means that the Id of such anti-Id molecules would result in a mimic of the Ag, as both interact with the same region (the paratope) on the target. Because of this relationship, the Ids of the restricted group of anti-id Abs that resemble the epitope of the Ag can be considered as a mimic of the original antigen. Consequently, when used as Ags, such anti-id Abs might induce a response in the host partially similar to that triggered by the specific epitope of the original Ag, as this is the only mimicked region.

The use of mimic Ags represents a valuable strategy in cases in which only Abs against a well-defined epitope possess functional/neutralizing activity, as it is the case of Abs effectively controlling flavivirus infections such as dengue virus (DENV), Zika virus (ZKV), and West-Nile virus (WNV). In these viruses, the most potent neutralizing Abs target the viral glycoprotein E (envelope), the main constituent of the viral membrane envelope, which is involved in host cell attachment and fusion to the endosome membrane during infection [3,4,5]. The mature E glycoprotein is assembled in anti-parallel dimers with a herringbone-like configuration that covers the surface of the virion [6,7].

Broad-spectrum and potent cross-neutralizing Abs conserved among different viruses and restricted to complex quaternary epitopes only present in the pre-fusion dimeric state of protein E (known as EDE, E dimeric epitopes) have been described and well characterized from DENV infected individuals. These epitopes are conserved among different DENV serotypes and partially in other flaviviruses [8,9,10,11]. In contrast, Abs targeting other epitopes on protein E have generally low neutralizing activity, with the exception of those directed towards Domain III (DIII), which are, however, virus and serotype-specific [12,13]. In addition, most low- or non-neutralizing Abs directed against other dominant epitopes on protein E, such as the fusion loop, largely contribute to the well-known Ab-dependent-enhancement (ADE) of infection [13,14]. Therefore, inducing an immune response restricted to EDEs through mechanisms of Ag mimicking is a potentially promising strategy, as it should induce exclusively anti-EDE neutralizing Abs with significantly reduced ADE. Here we describe the attempt to isolate, using a nanobody naive library [15], anti-id nanobodies against the human high-affinity monoclonal Ab (mAb) 1C10 [8] that specifically recognizes an EDE1 common to all DENV serotypes and Zika virus with the aim of using them as mimics of the EDE1 to induce neutralizing immune responses.

## 2. Materials and Methods

### 2.1. Preparation of the Ab Fragments

**Figure 1:**
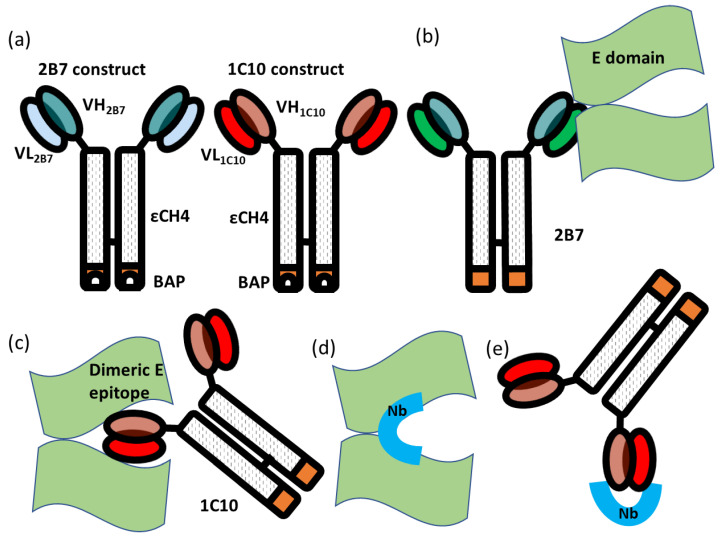
Representation of the biological material used for anti-idiotypic selection. (**a**) Graphical scheme of the Ab constructs exploited for the selection of nanobodies, VL and VH correspond to the variable domains of IgG light and heavy chains, respectively. Both 2B7 (and 1C8) and 1C10 are composed by the scFv domain of their corresponding original IgGs fused to an εCH4 domain (human IgE heavy chain constant domain 4) and a biotinylation sequence (Biotin Acceptor Peptide—BAP); (**b**) 2B7 and 1C8 (control constructs) bind to an epitope of the dimeric protein E domain of dengue 2 virus without inducing neutralization. (**c**) 1C10 (target construct) binds to a conserved epitope present in all dengue serotypes and Zika virus and induces neutralization. (**d**) Anti-idiotypic nanobodies (Nb) can structurally mimic the corresponding dimeric protein E domain epitope. (**e**) Their conformation enables specific binding to 1C10 paratope and, potentially, could induce 1C10-like immune response.

Fusion immunoproteins were built by cloning the single-chain variable fragment (scFv) sequences corresponding to the VH and VL regions of human IgGs specific for DENV in frame with those of the human εCH4 domain and a Biotin Acceptor Peptide tag for in vivo biotinylation catalyzed by co-expressed recombinant BirA [16]. Three mAbs were used, 2B7 and 1C8 (control clones), that bind to the virus without providing cross-neutralization, and 1C10, with potent neutralizing activity on all the dengue serotypes and Zika virus (described in [17,18] and depicted in Figure 1). When HEK293 cells were transfected with such plasmids, following the protocol previously described for the production of biotinylated EDIII antigens [12], they expressed and secreted biotinylated scFv-εCH4 Ab fragments that could be directly purified from dialyzed media using avidin/streptavidin. The same protocol [12] was used to produce dengue/Zika dimeric E epitopes.

### 2.2. Isolation of Nanobodies Specific for the 1C10 Construct

A pre-immune nanobody phage display library [15] was first depleted against the negative construct 2B7-εCH4. The library unbound fraction was successively panned against the target antigen 1C10-εCH4 (enrichment step). Briefly, 20 µg of mono-biotinylated 1C10 and 2B7 constructs were resuspended in 1 mL of PBS and then incubated with 100 µL of streptavidin-coated magnetic beads (Dynabeads MyOne T1, Invitrogen, Waltham, MA, USA) under rotation at 4 °C, overnight. The magnetic beads were washed according to the manufacturer’s recommendations and resuspended in 1 mL of blocking buffer (2% BSA in PBS). Simultaneously, 1 mL of blocking solution was added to a VHH library aliquot containing 10^12^ phages and the microtube was incubated for 30 min under rotation at 4 °C. Subsequently, the blocking solution of 2B7-coated beads (depletion tube) was discarded, the blocked library was transferred to the depletion tube and incubated for 1 h under rotation at RT. Finally, the supernatant corresponding to the depleted library was collected, transferred to the tube containing the blocked 1C10-coated beads, incubated under rotation for 2 h at RT and then washed 20 times with PBS + 0.05% Tween 20 (PBST). After the washing step, magnetic beads were recovered and resuspended in 900 µL of glycine 0.1 M, pH 2.2 to induce the elution of the bound phages. Buffer neutralization was performed after 10 min of incubation at RT by adding 250 µL of 1 M Tris-HCl, pH 9.1. The eluted phages were amplified using *E. coli* TG1 cells infected with M13KO7 helper phages before undergoing a second panning round. At the end of this, the specificity of the eluted phages for 1C10 was assessed by phage ELISA assay.

### 2.3. Identification of 1C10-Specific Binders

Phages corresponding to unique clones were recovered by infecting 9.25 mL of exponentially growing TG1 cells with 750 µL of the phage elution fraction obtained from the second panning round. After 30 min at 37 °C, the infected bacteria were centrifuged and the pellet was resuspended in 2TY medium to be plated immediately on 2TY agar + 1% glucose + ampicillin (100 µg/mL) and incubated at 37 °C, overnight. The grown bacteria were collected in 4 mL of 2TY medium supplemented with 30% glycerol. An aliquot of 50 µL was used for DNA extraction using the QIAprep Spin Miniprep kit (QIAGEN, Hilden, Germany). The DNA obtained was transformed into TG1 competent cells, plated on 2TY agar medium + 1% glucose + ampicillin (100 µg/mL) and incubated overnight at 37 °C. Afterwards, 92 isolated colonies were grown in deep-well microplates containing 1 mL of 2TY + 1% glucose + ampicillin (100 µg/mL) and incubated overnight at 37 °C under constant shaking (200 rpm). After incubation, 80 µL of the cultured bacteria were transferred to 1 mL of the same fresh medium and the deep-well plates were incubated at 37 °C. When the bacterial cultures reached the OD600nm of 0.5, they were infected with helper phages and incubated for 30 min at 37 °C without shaking. Then the plates were centrifuged, and the bacterial pellets were first resuspended in 1 mL of 2TY + ampicillin (100 µg/mL) + kanamycin (50 µg/mL) and successively incubated at 30 °C overnight under shaking. The plate was centrifuged, the phage-containing supernatants transferred to a sterile 96-well microplate and blocked with 2% of BSA in PBS for 1 h at RT (blocked phages).

Nunc Maxi Sorp Immuno-Plates (ThermoFisher-Nunc, Roskilde, Denmark) were pre-coated with 100 µL/well of avidin (5 µg/mL in carbonate/bicarbonate coating buffer) and incubated overnight at 4 °C. The next day, the avidin solution was discarded and the wells were blocked in PBS, 1% BSA and incubated for 1 h at RT. Subsequently, the plates were washed 3 times with PBST and 100 µL of the biotinylated Ags, namely the positive 1C10 construct and the negative control 2B7 used for the depletion step, was added to the plates and incubated overnight at 4 °C. On the third day, the wells were washed three times and 100 µL of blocked phages was added to both 1C10 and 2B7 containing wells before being incubated for 2 h at RT. The wells were then washed three times with PBST and 100 µL/well of anti M13 HRP-conjugated antibody (diluted 1:5000 in PBS plus 1% BSA) was added. The plates were incubated for 1 h at RT before being washed 5 times with PBS. The color reaction was developed in the presence of 3,3,5,5-Tetramethylbenzidine (TMB, Sigma, St. Louis, MO, USA) as a substrate and stopped with 2 N H_2_SO_4_. The absorbance was measured at 450 nm using a plate-reading spectrophotometer (Tecan, Männedorf, Switzerland). Only phage samples with OD450 nm values of the target 1C10 at least 5 times higher than the 2B7 control were considered positive. After such preliminary screening, putative positive samples were tested again in triplicate. Positive clones selected by phage ELISA were grown in 5 mL of LB medium + 100 mg/mL ampicillin and their plasmids were isolated using the QIAprep Spin Miniprep kit (QIAGEN) and sequenced using the M13Rv primer (5′-CAGGAAACAGCTATGACCATG-3′).

Bacteria transfected with phages displaying either the anti-idiotypic nanobodies specific for 1C10 or irrelevant binders (negative controls) were grown overnight in 2xTY medium supplemented with ampicillin and kanamycin. After phage purification and quantification, 96-well plates were coated overnight at 4 °C with 0.1 mg of phages resuspended in PBS plus 2% BSA before the addition of mouse sera diluted 1:100 in PBS. After two hours of incubation, mouse sera containing the antibodies 1C10 and 2B7 were incubated with the adsorbed nanobodies (both targets and negative controls) and the captured antibodies were detected using HRP-conjugated anti-mouse Fc γ-specific IgG antibodies (Jackson Immuno Research, Newmarket, UK) diluted 1:10,000. The sequences corresponding to 20 nanobody clones identified as positive for 1C10 during the screening were subcloned downstream of an Ig secretion leader and upstream of the human γCH3 domain in a pCDNA3 secretion vector. Sequence identity was verified by Sanger sequencing and Midi DNA preps were obtained using NucleoBond Xtra Midi Kit (Macherey-Nagel, Düren, Germany). The DNA was used to transfect HEK293T cells and the resulting VHH-γCH3 fusion constructs were recovered from the culture medium supernatant. Their binding efficacy was tested by flow cytometry and compared using a mock culture medium, stable cell lines displaying no scFv (Sp2/0 wt), negative control scFvs (Sp2/0-2B7_εCH4/SIP and Sp2/0-1C8_εCH4/SIP), and target scFv (Sp2/0-1C10_εCH4/SIP). Visualization was obtained with an anti-human IgG Alexa 488-conjugated. The expression level of the three displayed clones has been positively verified by means of an anti-human IgE. In parallel, the media from all transfected cells were assessed by ELISA to quantify their overall expression level. Plates were coated with 25 µL of the supernatants containing the VHH-γCH3 constructs and the detection was obtained by means of a goat anti-human IgG primary antibody and a rabbit anti-goat IgG secondary antibody conjugated with HRP. The color reaction was obtained using TMB as the substrate and quantified reading the OD at 450 nm.

### 2.4. Evaluation of the In Vivo Immune Response

Six-week-old female Balb/c mice were used for the in vivo vaccination test. Three mice were immunized with each of the 11 selected VHH clones (A11, B7, B10, C3, D10, D12, E6, G4, G5, G6, G10) and 2 non-immunized mice were used as the control group. The protocol foresaw three immunizations with 1 µg of DNA at day 0, 15, and 30 performed with a Gene Gun. Blood samples were collected at day 45 by sub-mandibular puncture and sera were stored at −20 °C. The evaluation of mouse immune response against the antigens human γCH3 and dengue/Zika dimeric E epitope was obtained by ELISA as described above using 100 µL of 3 µg/mL of human IgG (Sigma) and 100 µL of dialyzed dengue and Zika biotinylated antigens, respectively, for coating. The antibody 1C10 (assay positive control) was used diluted 1:100. Statistical analysis was performed using a 2-sided analysis of variance (ANOVA), 95% confidence level, with Tukey’s pairwise comparison at 95% confidence.

The immune response elicited by the anti-idiotypic VHH was assessed by ELISA. Nunc MaxiSorp Immunoplates (ThermoFisher-Nunc) were coated with 100 μL/well of 5 μg/mL avidin (Sigma) in 50 mM Na_2_CO_3_/NaHCO_3_ buffer pH 9.5 and incubated overnight at 4 °C. Plates were washed three times in PBST buffer (0.05% Tween 20 in PBS pH 7.4), blocked with 1% BSA in PBST for 1 h at room temperature, and coated with the dialyzed biotinylated antigens (A11, E6 and G5) diluted in PBS (10 ng in 100 μL/well), at 4 °C overnight. Plates were washed three times in PBST buffer and incubated with 2xfold mouse sera dilutions (1:100) in PBST + 1% BSA for 2 h at room temperature. HRP-labelled goat anti-mouse IgG (Jackson Immuno Research, Cambridge, UK) diluted 1:30,000 in PBST + 1% BSA was added and incubated for 1 h at room temperature. Substrate solution (TMB) was added in each well (50 μL/well), the reaction was stopped with H_2_SO_4_ 1 M (50 μL/well) and OD450 was measured on a micro-plate reader (Tecan).

## 3. Results

**Figure 2:**
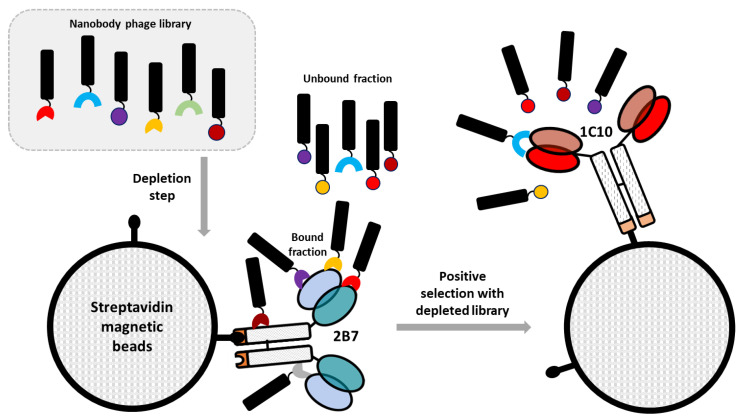
Panning strategy. The phage library was first depleted against the irrelevant biotinylated 2B7 construct and the unbound phage fraction (depleted library) was used for the selective biopanning against the 1C10 target construct. Both 2B7 and 1C10 are immobilized on streptavidin magnetic beads.

The aim of the present research was the identification of a set of binders selective for the idiotype of a monoclonal antibody (1C10) with neutralizing activity against DENV that specifically recognizes the EDE1 of the E glycoprotein (Figure 1). The rationale behind this strategy is that their paratope should, in some cases, correspond to the complementary structure of the original antibody paratope, resembling the structure of the original antigen. This characteristic provides the theoretical opportunity that such anti-idiotypic nanobodies might elicit an effective immune response when used as immunization antigens. Thus, a biopanning strategy was conceived to isolate nanobodies specific for mAb 1C10 (Figure 2).

To this aim, a naïve phage-displayed nanobody library was initially depleted against a structurally similar mAb (2B7) that recognizes an epitope on the dimeric E (EDE2), which is different from EDE1. The constructs used for the selection were composed by a scFv containing the antibody variable VL and VH domains fused to the human ɛCH4 domain ([16], Figure 1a). This step was foreseen to eliminate all those clones binding to “background epitopes”, namely epitopes shared between depletion and target antibodies (Figure 2). Successively, the unbound library fraction was challenged with a construct containing the scFv of the 1C10 mAb. After washing out unspecific phages, the idiotype-binding nanobodies were eluted, amplified, and subjected to another round of biopanning using the same protocol. Given the panning strategy, the resulting nanobodies should have the characteristic of being specific for the 1C10 variable regions and therefore with anti-idiotypic activity (Figure 1e). The binding specificity was tested by phage ELISA performed in triplicate on both constructs 2B7 (depletion) and 1C10 (specific). The initial screening was carried out on 92 clones isolated from the output of the second round of panning and identified 39 clones classified as positive because the ratio between the signals obtained with 1C10 and 2B7 was higher than five. After DNA sequencing, 27 unique sequences were identified and aligned (Supplementary Material Figure S1). Since the naïve library used as nanobody source was created by pooling the genetic material from 20 animals [15], we were not surprised to have identified conserved sequences differing only for few residues but possessing high consensus in the CDRs that usually represent most of the nanobody paratope [19]. Inside each of these clusters, the best representatives according to the ELISA results were selected for the successive step together with the unique sequences, for a total of 20 individual clones (Supplementary Material Figure S1).

**Figure 3:**
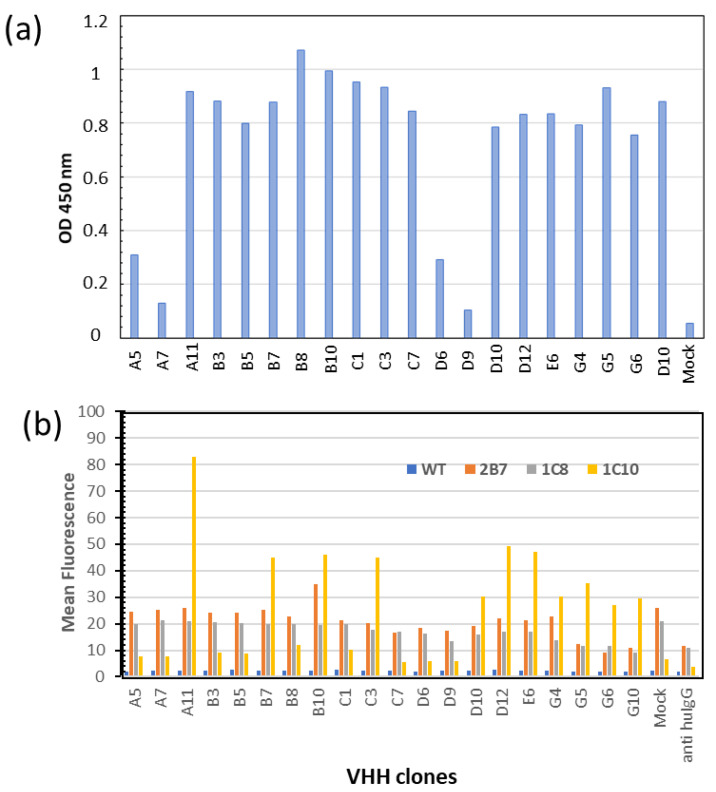
Characterization of the nanobodies expressed in mammalian cells as fusions with human γCH3 domain and selection of clones to be used as vaccines. (**a**) Quantification of the secreted constructs by ELISA performed using 25 µL of supernatant; (**b**) their binding specificity for the antigen 1C10 was evaluated by flow cytometry and compared with the binding to the irrelevant Abs 2B7 and 1C8 that share the same structure but differ for their CDRs. WT corresponds to the empty vector.

**Figure 4:**
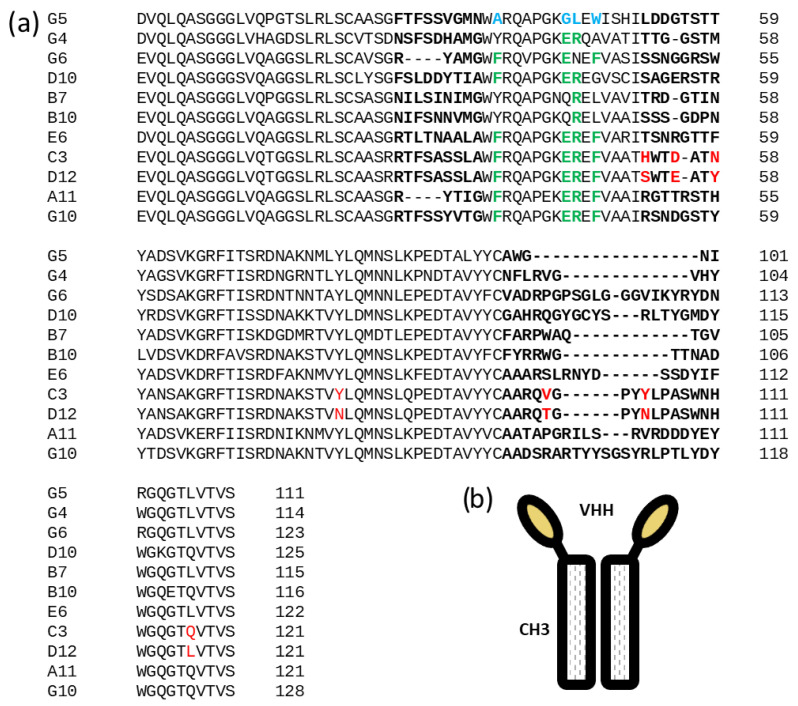
Sequence alignment of 1C10-specific Nbs. (**a**) List of the clones validated as anti-idiotypic towards 1C10 and selected as antigens for in vivo experiments. CDRs are in bold, green letters identify hallmark residues of camelid nanobodies (VHH), blue letters hallmark residues of conventional VH and red letters single mutations between conserved sequences. (**b**) Schematic representation of the immune construct used as the anti-1C10 reagent and in vivo antigen composed of two human γCH3 domains and two nanobody domains.

The corresponding sequences were sub-cloned and expressed as VHH-γCH3 fusion proteins secreted from mammalian cells [12]. Among the 20 constructs, 16 were secreted with similar efficiency in the culture media of transfected HEK293T cells (Figure 3a). Their binding specificities were then tested by flow cytometry on cells’ membrane—displaying either the specific target scFv 1C10 or the negative controls scFvs 2B7 or 1C8 [11]. Eleven clones showed high and specific binding to 1C10 (Figure 3b). Their sequences are reported in Figure 4a. Surprisingly, clones A5 and C1 that had the highest scores in the initial screening phage ELISA (Supplementary Material Figure S1) did not pass this step in which the binders were expressed as VHH-γCH3 fusions (Figure 4b). We noticed that these clones were the only to possess an extra (third) cysteine in the framework 4 of their sequences. Such residues might be involved in the formation of intermolecular disulfide bonds in oxidizing environments and the higher avidity of the resulting dimers could have affected the screening ELISA results.

All clones possessed the typical (or common conservative alternatives) camelid hallmarks at the residues 37, 44, 45, and 47 of the framework 2 (green letters in Figure 4a), with the exception of G5. This clone represents a particularly interesting case because its sequence possesses the hallmarks of a conventional VH (blue letters in Figure 3b). The identification of such odd sequences among functional nanobodies is not rare in natural llama repertoire [20] and their amino acid combination has been exploited to obtain functional and stable humanized nanobodies [21].

Next, all the selected clones were tested for their suitability as antigens by means of in vivo experiments in mice. DNA immunization by gene-gun was carried out using the 11 VHH-γCH3 plasmids on three animals per construct. The immune response induced by the anti-idiotypic nanobodies was first evaluated by ELISA on phage-coated plates (Supplementary Material Table S1). In most of the cases (9 out of 11) sera were able to recognize nanobodies, with 5 of them (A11, C3, D10, D12, G4) showing specificity for the variable regions, namely the nanobody CDRs, while the others showed either cross-reactivity (B7, E6, G8, G10), indicating binding to conserved framework epitopes, or no response (B10, G5). Furthermore, all animal sera reacted against the human γCH3 domain, thus confirming that immunization was successful in all cases (Supplementary Material Table S2). Sera from animals immunized with three selected nanobodies belonging to specific (A11), cross-reactive (E6), and negative (G5) groups were then analyzed by ELISA on the immunizing antigens expressed from mammalian cells (Table 1). The results confirmed that while antibodies induced by VHH A11 were rather specific with anti-idiotypic characteristics, G5 elicited only a negligible immune response and the antibodies induced by E6 recognized epitopes conserved among all three tested nanobodies, and therefore probably belonging to their framework regions.

To test whether any of the 11 nanobodies used as antigens were able to mimic the EDE1 epitope, sera were then tested for their ability to recognize the DENV dimeric E protein by ELISA. Regrettably, none of them were able to bind to the viral E protein (Supplementary Material Figure S2). Altogether, the data illustrated the large diversity of immune response that the anti-idiotypic nanobodies elicited in vivo. At the same time, they indicated that the selected anti-idiotypic nanobodies failed to elicit a selective, neutralizing reactivity towards the EDE1 epitope on the E dimer, despite the fact that the induced antibodies can target specifically the nanobody CDRs, that should contribute to most of the anti-idiotypic structures.

## 4. Discussion

Anti-idiotypic Nbs have been largely used to replace toxins in immunoassays with the aim to ameliorate operator and environmental safety, improve assay standardization, and reduce costs [22,23]. The conventional strategy used for their isolation consists in using an available IgG specific for the antigen as the target for panning a pre-immune library, as in the case of nanobodies used to quantify fumonisin B1 contaminations in cereals [24]. The structurally related Ab fragments of shark origin (vNARs) as well as the small alternative scaffold of affimers seem also suitable for isolating anti-idiotypic binders specific for IgG paratopes starting from synthetic/semi-synthetic libraries. They have been exploited to obtain reagents selective for therapeutic Abs [25,26,27] that could be employed, for instance, for pharmacokinetic studies and to detect the concentration of circulating Abs in treated patients. In these cases, it was not evaluated whether anti-idiotypic binders were mimics of the antigens, despite anti-idiotypic Abs can be exploited as immunogenic factors to induce an epitope-specific response. Alvarez-Rueda et al. [28] demonstrated the feasibility of using a nanobody raised against the trastuzumab paratope to induce in immunized mice the generation of anti-HER2 Abs able to inhibit the viability of HER2-positive cells.

Nevertheless, no further article reporting the generation of anti-idiotypic nanobodies able to induce effective immune response in vivo has appeared since 2009 and this observation might indicate that successful conditions are difficult to match. From a statistical point of view, our attempt was promising since the panning results were more favorable than those described by Alvarez-Rueda who immunized a llama with the trastuzumab Fab fragment, prepared an immune nanobody library but recovered only a single potential candidate after ELISA screening. However, this single clone resulted in an immunogenic and induced target-specific response. We panned a pre-immune library applying a very stringent in vitro depletion step and isolated 27 clones that, according to ELISA test, were selective for the idiotype of the neutralizing antibody. This pool was reduced to 11 strong and highly expressed binders after a further screening performed by flow-cytometer on cells displaying native-like structures of the antigen. The extremely large variety of the nanobody CDRs of the validated clones was another favorable element because it corresponded to highly divergent nanobody paratopes and, consequently, to several surfaces available for eliciting alternative immune responses. Therefore, the lack of immune response specific for the EDE1 on the viral protein E in all mice was somehow deceiving, considering that the animals strongly reacted not only against the conserved γ-CH3 domain and nanobody framework, but in several cases also against the nanobody variable region formed by the CDRs. The most logical explanation we found points to the structural characteristics of the epitopes. Despite trastuzumab epitope seems quite complex, works performed with single linear peptides inside the overall epitope region showed that mouse immunization with one of them was already sufficient to elicit a protective immune response against HER2-positive cell proliferation [29]. The system seems relatively tolerant, since protective immune response can be triggered also by peptides that do not match exactly the HER2 sequence [30]. This suggests that an anti-idiotypic nanobody mimicking a single linear (sub)-epitope could induce a protective immunogenic response in treated animals. In the case of the dengue virus protein, diagnosis can be effectively performed with Abs and nanobodies against viral protein NS1 that have variable cross-reactivity with respect to the four serotypes [31,32,33]. However, only Abs targeting the conformational epitope formed by the dimerization of the E domain possess high neutralizing activity and reduced ADE. Structurally, this epitope is probably more complex and consequently more difficult to mimic in a physiologically useful manner than a linear amino acid sequence. Our results show that at least the A11 clone (and probably the other four clones with similar reaction patterns reported in Supplementary Material Table S2) seems to induce an immune response specific for its paratope but its strong binding to the 1C10 variable region apparently involves a surface that does not match sufficiently the antigen structure inducing neutralizing response. In other words, it triggers an immune response that is anti-idiotypic but not a perfect mimic of the viral epitope and therefore does not provide neutralizing activity. Interestingly, we did not observe a more pronounced anti-CDR immune response in G5, despite it possessing a human/mouse-like fingerprint that should minimize the reactivity against its framework regions. Altogether, the data indicate that probably it would be necessary to test a significantly higher number of clones to identify the rare conformation able to elicit the desired immune response. They suggest as well that vaccination methods that are extremely effective in inducing immune reaction, such as those based on RNA delivery, would not be automatically successful when applied to dengue/zika viruses. This is because the vaccination efficacy against these viruses depends on the availability of a highly specific and complex conformational epitope rather than a larger macrodomain. The experimental data we obtained with anti-idiotypic nanobodies possessing different sequences will help to integrate the ex novo computation modeling that we undertook with the aim to design in silico epitopes able to trigger neutralizing response.

## 5. Conclusions

Our work confirms that in vitro selection of nanobodies performed imposing stringent parameters represents a valuable modality to isolate binders specific for particular domains, such as antigen epitopes and Ab paratopes [34,35]. We obtained a large and structurally highly heterogeneous array of clones with specificity for a specific idiotype. Nevertheless, this variety was insufficient to isolate binders functionally suitable for vaccination. Epitope mapping performed by hydrogen–deuterium exchange mass spectrometry was successfully exploited to identify antibody-bound epitopes [36] and might provide the information necessary to identify the antigen key residues that trigger selective immune response. This knowledge could guide the in silico transformation of anti-idiotypic hits, targeting inappropriate regions of the neutralizing paratope, into leads able to elicit functional response.

## Figures and Tables

**Table 1:** Mouse immune response to nanobody vaccination measured by ELISA. Sera from immunized mice (three animals for each nanobody clone, indicated as serum 1, 2, and 3, respectively) were tested against the corresponding antigens (nanobodies A11, E6, G5). Sera were also allowed to cross-react with different anti-idiotypic nanobodies as well as an irrelevant nanobody (control). As controls, pre-immune serum, the blocking reagent BSA, and the reaction mix without sera were used. Results correspond to OD values measured at 450 nm and were expressed as means of ± standard deviation (SD).

Immunized Mice	Nanobodies			
	A11	E6	G5	Control
A11 serum1	**1.07 ± 0.08**	0.29 ± 0.04	0.09 ± 0.01	0.11 ± 0.02
A11 serum2	1.13 ± 0.15	-	-	0.10 ± 0.01
A11 serum3	1.02 ± 0.19	-	-	0.13 ± 0.01
E6 serum1	1.13 ± 0.01	1.04 ± 0.17	1.16 ± 0.02	1.01 ± 0.05
E6 serum2	-	0.98 ± 0.11	-	0.94 ± 0.04
E6 serum3	-	1.24 ± 0.06	-	0.99 ± 0.07
G5 serum1	0.38 ± 0.02	0.39 ± 0.04	0.77 ± 0.03	0.32 ± 0.03
G5 serum2	-	-	0.67 ± 0.01	0.26 ± 0.01
G5 serum3	-	-	0.31 ± 0.01	0.30 ± 0.02
Pre-immune serum	0.07 ± 0.01	0.08 ± 0.01	0.07 ± 0.01	0.10 ± 0.02
BSA	0.06 ± 0.01	0.06 ± 0.01	0.06 ± 0.01	0.05 ± 0.01
No sera	0.06 ± 0.01	0.05 ± 0.01	0.05 ± 0.01	0.06 ± 0.02

## Data Availability

Not applicable.

## Supplementary Materials

The following supporting information can be downloaded at: https://www.mdpi.com/article/10.3390/biom13030551/s1, Figure S1: Alignment of sequences corresponding to potential binders of 1C10 CDRs; Figure S2: ELISA to test serum reactivity against Dengue CVD-Envelope; Table S1: Mouse immune response towards nanobody structural components; Table S2: Mouse response specific for Human γ-CH3.
