# Supplementary material for: Nanobodies Selectively Binding to the Idiotype of a Dengue Virus Neutralizing Antibody Do Not Necessarily Mimic the Viral Epitope
Source: Biomolecules. 2023 Mar 17;13(3):551. doi: 10.3390/biom13030551 (PMC10046864; doi:10.3390/biom13030551)
Supplement: Supplementary file 1 [file biomolecules-13-00551-s001.zip › biomolecules-2189854-supplementary.pdf]

**Supplementary Materials**

**Figure S1.** Alignment of sequences corresponding to potential binders of 1C10 CDRs. **Ratio*** indicates the ratio of $Abs_{450nm}$ obtained by testing in ELISA the clones against the target 1C10 and the depletion 2B7 constructs, respectively. ELISA was performed in triplicates. Unique sequences (27) with a ratio value > 5 were aligned and conserved sequences grouped into clusters. Amino acids corresponding to CDR3 have been highlighted. The best representatives of each cluster, according to the ELISA results, were selected for the successive step together with the unique sequences, for a total of 20 individual clones, highlighted in red.

**CLUSTER 1** **Ratio***

D6 MADVQLQASGGGLVQAGGSLRLSCAASGSTFSIDWFGWYRQAPRKQRESVATVSTTGDTD (26,6)
B8 MADVQLQASGGGLVQAGESLRLSCTASESTFSFHPFGWYRQAPGKPRELVATVSRYGDTY (15,4)
E7 MAEVQLQASGGGLVQAGESLRLSCTASESTFSFHPFGWYRQAPGKPRE*VATVSKYGDTY (16,0)
A7 MAEVQLQASGGGLVQAGGSLRLSCVSSGSTFSVHHFGWYRQAPGKQRQTVATVTTFGDTY (28,1)
B5 MAEVQLQASGGGLVQAGGSLRLSCASSGSTFSVHAFGWYRQAPGKQRETVATVTKTGDTY (52,9)

D6 YADSVNGRFTVSTDNNERSVYLRMDTLHPDDSAVYDC**SAGAGRYTDFW**GQGTLITVS
B8 YADSVVGRFTISRDDSMSTVYLQMNSLKPEDSAVYYC**YAGVAMYLDAW**GQGTQVTVS
E7 YADSVVGRFTVSRDDSRSTVDLQMNSLKPEDSAVYYC**YAGVAMYLDAW**GQGTQVTVS
A7 YADSVKGRFTVSRDDNKSAVYLQMDNLQPEDTAVYYC**YIGAAMYDDYC**RQRTVITVS
B5 YADSVKGRFTISRDDNKNTVYLQMNNLQPEDTAVYYC**YTGAAMYDDYC**GQSNLIIVS

**CLUSTER 2**

B1 MADVQLQASGGGLVHAGGSLRLSCAASGHTLTNAALAWFRQAPGKEREFVARITSNRGTT (38,3)
E6 MADVQLQASGGGLVQAGGSLRLSCAASGRTLTNAALAWFRQAPGKEREFVARITSNRGTT (10,6)
D11 MAEVQLQASGGGLVQAGGSLRLSCAASGRTLTNAALAWFRQAPGKEREFVARITSNRGTT (10,6)
H7 MAEVQLQASGGGLVQAGGSLRLSCAASGRTLTNAALAWFRQAPGKEREFVARITSNRGTT (9,8)

B1 FYAYSVKDRFTISRDFAKNMVYLLMNSLKFEDTAVYYC**AAARSLRNYDSSDYIFW**GQGTQ
E6 FYADSVKDRFTISRDFAKNMVYLQMNSLKFEDTAVYYC**AAARSLRNYDSSDYIFW**GQGTL
D11 FYADSVKDRFTISRDFAKNMVYLQMNSLKFEDTAVYYC**AAARSLRNYDSSDYIFW**GQGTL
H7 FYADSVKDRFTISRDFAKNMVYLQMNSLKFEDTAVYYC**AAARSLRNYDSSDYIFW**GQGTQ

B1 VTVS
E6 VTVS
D11 VTVS
H7 VTVS

**CLUSTER 3**

D12 MAEVQLQASGGGLVQTGGSLRLSCAASRRTFSASSLAWFRQAPGKEREFVAATSWTEATY
(18,8)
C3 MAEVQLQASGGGLVQTGGSLRLSCAASRRTFSASSLAWFRQAPGKEREFVAATHWTDATN
(14,6)
F1 MAEVQLQASGGGLVQTGGSLRLSCAASRRTFSASSLAWFRQAPGKEREFVAATHWTDATN
(7,9)
H3 MADVQLQASGGGLVQTGGSLRLSCAASRRTFSASSLAWFRQAPGKEREFVAATHWTDATN
(6,2)

D12 YANSAKGRFTISRDNAKSTVNLQMNSLQPEDTAVYYC**AARQTGPYNLPASWNHW**GQGTLV
C3 YANSAKGRFTISRDNAKSTVYLQMNSLQPEDTAVYYC**AARQVGPYYLPASWNHW**GQGTQV
F1 YANSAKGRFTISRDNAKSTVYLQMNSLQPEDTAVYYC**AARQVGPYYLPASWNHW**GQGTQV
H3 YANSAKGRFTISRDNAKSTVYLQMNSLQPEDTAVYYC**AARQVGPYYLPASWNHW**GQGTQV

D12 TVS
C3 TVS
F1 TVS
H3 TVS

**CLUSTER 4**

C7 MAEVQLQASGGGLVQPGGSLRLSCSVSGNILSVNIMGWYRQAPGNQRELVAVITSDSTIN
(19,1)
B7 MAEVQLQASGGGLVQPGGSLRLSCSASGNILSINIMGWYRQAPGNQRELVAVITRDGTIN (7,0)
F10 MADVQLQASGGGLVQPGGSLRLSCSASGNILSINIMGWYRQAPGNQRELVAVITRDGTIN (6,7)

C7 YADSVKGRFTISKDSDMRTVYLQMDTLEPEDTGVYYCY**ARPWAQTCVW**GQRTHVIVS
B7 YADSVKGRFTISKDGDMRTVYLQMDTLEPEDTAVYYCF**ARPWAQTGVW**GQGTLVTVS
F10 YADSVKGRFTISKDGDMRTVYLQMDTLEPEDTAVYYCF**ARPWAQTGVW**GQGTQVTVS

**UNIQUE SEQUENCES**

D9 MADVQLQASGGGLVQTGGSLRLSCAASGRTFSISSLGWFRQAPGKEREFVAATSWTD-AT
(31,0)
B3 MADVQLQASGGGLVQAGGSLRLSCAASGSIFSDYVMGWYRQAPGNQRELVATITADG-WM
(60,4)
A5 MADVQLQASGGGLVQPGGSLRLSCTVSGTIFTANDMGWYRQAPGKQRQAVALITTTT-DT
(125)
G5 MADVQLQASGGGLVQPGTSLRLSCAASGFTFSSVGMNWARQAPGKGLEWISHILDDGTST
(14,2)
G4 MADVQLQASGGGLVHAGDSLRLSCVTSDNSFSDHAMGWYRQAPGKERQAVATITTG-GST
(17,7)
G6 MAEVQLQASGGGLVQAGGSLRLSCAVSGR----YAMGWFRQVPGKENEFVASISSNGGRS
(17,3)
D10 MAEVQLQASGGGSVQAGGSLRLSCLYSGFSLDDYTIAWFRQAPGKEREGVSCISAGERST
(29,1)
B10 MAEVQLQASGGGLVQAGGSLRLSCAASGNIFSNNVMGWYRQAPGKQRELVAAISSSGD-P
(84,7)
C1 MADVQLQASGGGLVQAGGSLRLSCAASGRTSSNNHMGWFRQAPGKEREFVAAISWSGNRT
(91,1)
A11 MAEVQLQASGGGLVQAGGSLRLSCAASGR----YTIGWFRQAPEKEREFVAAIRGTTRST
(28,8)
G10 MAEVQLQASGGGLVQAGGSLRLSCAASGRTFSSYVTGWFRQAPGKEREFVAAIRSNDGST
(14,0)

D9 YYADSAKGRFTISRDDDMSTVNLQMNSLQPEDTAVYYC**SARPTG------------HYDL**
B3 KYADSVKGRFSISTDDDMNTVSLQMNSLKLEDTAVYYC**YARY---------SPAIYGDTY**
A5 TYANSVKGQFTISRDNSKNTVYLLMISLKPDDTGVYYC**KILP------------VGDDY**
G5 TYADSVKGRFITSRDNAKNMLYLQMNSLKPEDTALYYC**AWGN----------------I**
G4 MYAGSVKGRFTISRDNGRNTLYLQMNNLKPNDTAVYYC**NFLR------------VGVHY**
G6 WYSDSAKGRFTISRDNTNNTAYLQMNNLEPEDTAVYFC**VADRPGPSGLGG-GVIKYRYDN**
D10 RYRDSVKGRFTISSDNAKKTVYLDMNSLKPEDTAVYYC**GAHRQGYGCY---SRLTYGMDY**
B10 NLVDSVKDRFAVSRDNAKSTVYLQMNSLKPEDTAVYFC**FYRR-----------WGTTNAD**
C1 YYSDSVKGRFIISRDNAKNTVYLLMNSLKPDDTAVYYC**AAHSSIAALERP-SRTVDEYDY**
A11 HYADSVKERFIISRDNIKNMVYLQMNSLKPEDTAVYVC**AATAPGRILS---RVRDDDYEY**
G10 YYTDSVKGRFTISRDNAKNTVYLQMNSLKPEDTAVYYC**AADSRARTYYSGSYRLPTLYDY**

D9 **W**GPWTH****
B3 **W**SQGTVVSVS
A5 **C**DPANHVSFS
G5 **R**GQGTLVTVS
G4 **W**GQGTLVTVS

| | |
|---|---|
| G6 | **R**GQGTLVTVS |
| D10 | **W**GKGTQVTVS |
| B10 | **W**GQETQVTVS |
| C1 | **C**GQGTLVTDS |
| A11 | **W**GQGTQVTVS |
| G10 | **W**GQGTQVTVS |

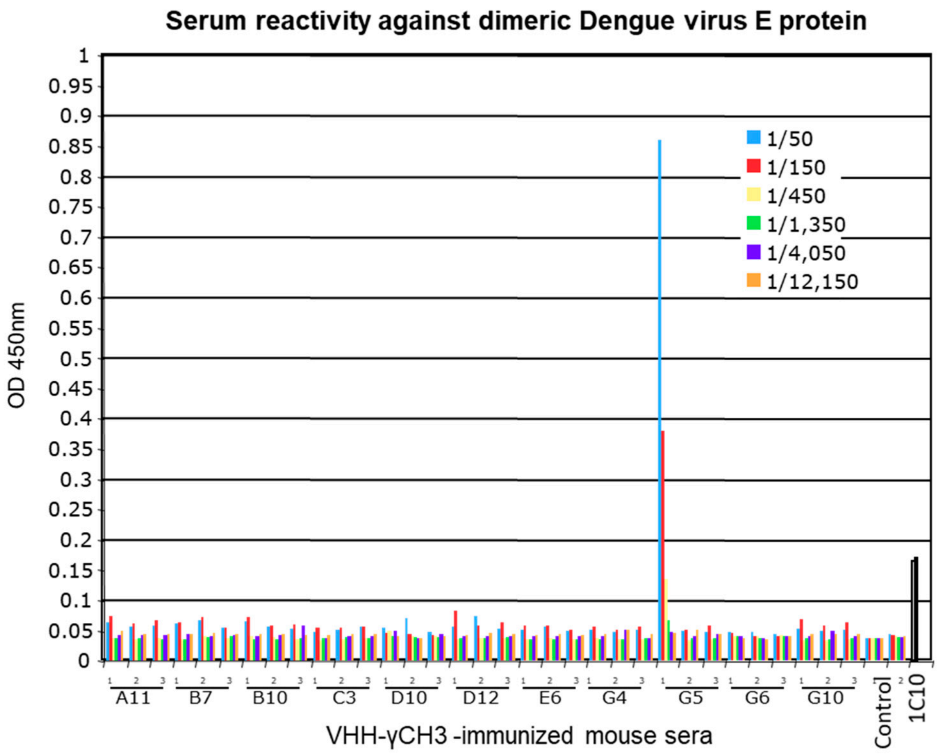


**Figure S2.** ELISA to test serum reactivity against Dengue CVD-Envelope. No sera showed specific activity. The only apparently positive signal detected in the experiments resulted being not specific.

**Table S1.** Mouse immune response towards nanobody structural components measured by ELISA. Each of the different antigens (anti-idiotypic nanobodies) was used to immunize tree mice and their sera were tested against the specific nanobody used for their immunization (specific antigen) as well as against two irrelevant nanobodies used as controls (negative controls). The rationale is that cross-reacting sera positive with any nanobody (red) recognize conserved epitope, probably belonging to the nanobody framework; non-reactive/weak-reactive sera are apparently poorly antigenic (blue); sera specific only for the nanobody used as antigen (green) should recognize the variable nanobody regions (CDRs).

| Mice | Nanobodies | | |
|---|---|---|---|
| | Specific antigen | Negative control 1 | Negative control 2 |
| **A11.1** | 0.97 | 0.16 | 0.21 |
| **A11.2** | 0.98 | 0.19 | 0.11 |
| **A11.3** | 0.96 | 0.13 | 0.14 |
| **B7.1** | 0.71 | 0.68 | 0.56 |
| **B7.2** | 0.79 | 0.82 | 0.89 |
| **B7.3** | 0.88 | 0.80 | 0.83 |
| **B10.1** | 0.36 | 0.31 | 0.30 |
| **B10.2** | 0.24 | 0.20 | 0.18 |
| **B10.3** | 0.30 | 0.31 | 0.26 |
| **C3.1** | 0.94 | 0.18 | 0.12 |
| **C3.2** | 0.80 | 0.14 | 0.10 |
| **C3.3** | 0.82 | 0.19 | 0.25 |
| **D10.1** | 0.95 | 0.16 | 0.20 |
| **D10.2** | 0.95 | 0.18 | 0.16 |
| **D10.3** | 0.88 | 0.29 | 0.27 |
| **D12.1** | 0.74 | 0.26 | 0.21 |
| **D12.2** | 0.91 | 0.11 | 0.19 |
| **D12.3** | 0.94 | 0.67 | 0.15 |
| **E6.1** | 0.93 | 0.89 | 0.93 |
| **E6.2** | 0.92 | 0.92 | 0.84 |
| **E6.3** | 0.89 | 0.91 | 0.86 |
| **G4.1** | 0.88 | 0.17 | 0.18 |
| **G4.2** | 0.88 | 0.18 | 0.21 |
| **G4.3** | 0.82 | 0.21 | 0.12 |
| **G5.1** | 0.44 | 0.27 | 0.16 |
| **G5.2** | 0.40 | 0.32 | 0.27 |
| **G5.3** | 0.26 | 0.24 | 0.22 |
| **G8.1** | 0.69 | 0.68 | 0.70 |
| **G8.2** | 0.68 | 0.64 | 0.72 |
| **G8.3** | 0.75 | 0.64 | 0.75 |
| **G10.1** | 0.82 | 0.84 | 0.78 |
| **G10.2** | 0.85 | 0.86 | 0.36 |
| **G10.3** | 0.82 | 0.86 | 0.88 |

Summarizing, there are three kinds of serum reactivity towards the antigens: cross-reactive response (positive for any nanobody); no response (negative for all nanobodies); specific for the nanobody used for immunization (positive for the nanobody variable region corrsponding to the CDRs).

| | | | |
|---|---|---|---|
| **Cross-reactive** | **pos** | **pos** | **pos** |
| **anti-CDRs** | **pos** | **neg** | **neg** |
| **no response** | **neg** | **neg** | **neg** |

**Table S2**. Mouse serum reactivity against the human γ-CH3 domain fused to the nanobodies used for mouse immunization. ELISA results were obtained by reacting mouse sera with immobilized target nanobodies; pre-immune serum and PBS buffer were used as the negative controls. Three mice were analyzed for each antigen.

| | | Serum reactivity | |
|---|---|---|---|
| Nanobody clones | Mouse 1 | Mouse 2 | Mouse 3 |
| A11 | 0,919 | 0,94 | 0,927 |
| B7 | 0,895 | 0,873 | 0,84 |
| B10 | 0,926 | 1,025 | 0,788 |
| C3 | 0,919 | 0,938 | 1,032 |
| D10 | 0,878 | 0,874 | 0,841 |
| D12 | 0,988 | 1,03 | 0,891 |
| E6 | 0,973 | 0,96 | 0,982 |
| G4 | 0,935 | 1,065 | 1,002 |
| G5 | 0,889 | 0,784 | 0,953 |
| G6 | 0,861 | 1,056 | 1,092 |
| G10 | 0,802 | 0,894 | 0,98 |
| Pre-Immune serum | 0,038 | 0,034 | 0,033 |
| PBS | 0,039 | 0,038 | 0,038 |
